# Prevalence and prognostic value of malnutrition in patients with acute coronary syndrome and chronic kidney disease

**DOI:** 10.3389/fnut.2023.1187672

**Published:** 2023-07-14

**Authors:** Weicheng Ni, Kun Guo, Sanling Shi, Ling Cheng, Yimin Zhou, Fengyu Zhang, Jiachen Xu, Ken Lin, Changxi Chen, Zhan Gao, Hao Zhou

**Affiliations:** Department of Cardiology, The First Affiliated Hospital of Wenzhou Medical University, Wenzhou, China

**Keywords:** acute coronary syndrome, chronic kidney disease, malnutrition, major adverse cardiovascular events, all-cause mortality, global registry of acute coronary events score

## Abstract

**Background:**

Malnutrition is a rising global health issue associated with unfavorable outcomes of a variety of disorders. Currently, the prevalence and prognostic significance of malnutrition to patients with acute coronary syndrome (ACS) and chronic kidney disease (CKD) remained largely unclear.

**Methods:**

A total of 705 patients diagnosed with ACS and CKD in the First Affiliated Hospital of Wenzhou Medical University between 2013 and 2021 were included in this retrospective cohort study. Malnutrition was assessed by the Controlling Nutritional Status (CONUT), the Geriatric Nutritional Risk Index (GNRI), and the Prognostic Nutritional Index (PNI), respectively. The relationships between malnutrition and all-cause mortality and major cardiovascular events (MACEs) were analyzed.

**Results:**

During a median follow-up of 31 months, 153 (21.7%) patients died, and 165 (23.4%) had MACEs. The prevalence of malnutrition was 29.8, 80.6, and 89.8% for the PNI, CONUT, and GNRI, respectively. All the malnutrition indexes were correlated with each other (*r* = 0.77 between GNRI and PNI, *r* = −0.72 between GNRI and CONUT, and *r* = −0.88 between PNI and CONUT, all *p* < 0.001). Compared with normal nutrition, malnutrition was independently associated with an increased risk for all-cause mortality (adjusted hazard ratio for moderate and severe degrees of malnutrition, respectively: 7.23 [95% confidence interval (CI): 2.69 to 19.49] and 17.56 [95% CI: 5.61 to 55.09] for the CONUT score, 2.18 [95% CI: 0.93 to 5.13] and 3.16 [95% CI: 1.28 to 7.79] for the GNRI, and 2.52 [95% CI: 1.62 to 3.94] and 3.46 [95% CI: 2.28 to 5.25] for the PNI score. *p* values were lower than 0.05 for all nutritional indexes, except for moderate GNRI *p* value = 0.075). As for MACEs, similar results were observed in the CONUT and PNI. All the risk scores could improve the predictive ability of the Global Registry of Acute Coronary Events (GRACE) risk score for both all-cause mortality and MACEs.

**Conclusion:**

Malnutrition was common in patients with ACS and CKD regardless of the screening tools used, and was independently associated with all-cause mortality and MACEs. Malnutrition scores could facilitate risk stratification and prognosis assessment.

## 1. Introduction

Chronic kidney disease (CKD) as a strong risk factor for cardiovascular disease (1) affect approximately 30–40% of patients with acute coronary syndrome (ACS) (2). Though advanced medicine has been achieved through invasive procedures and pharmacological therapy, patients with ACS and CKD face a high risk of mortality and major adverse cardiovascular events (MACEs) (3). However, patients with advanced CKD have been widely excluded from clinical trials of cardiovascular interventions, which may be owing to cautiousness in certain conditions (hyperkalemia, anticoagulation, and high mortality) (4, 5). The performance of existing risk score, the Global Registry of Acute Coronary Events (GRACE) score, was impaired when predicting the prognosis of patients combined with dialysis (6, 7). It is necessary for physicians to identify high-risk patients based on modifiable clinical factors such as malnutrition to guide treatment and improve the prognosis of patients with ACS and CKD.

Malnutrition was found to be associated with poor prognosis of cancer, kidney disease, heart failure, and acute coronary syndrome (8–13). Malnutrition is a controllable risk factor based on which doctors could intervene, but the screening of it is often overlooked in clinical setting. Malnutrition screening is crucial because patients who are at risk may benefit from clinical nutritional therapy. The controlling nutritional status (CONUT) score (14), geriatric nutritional risk index (GNRI) (15), and prognostic nutritional index (PNI) (16) are tools that are comparatively easy to use and practical in clinical environment for screening malnutrition. Malnutrition, which is common among patients with ACS and patients with CKD, has been found to play an important role in the interaction between the heart and kidneys (9, 11, 17). However, the association between malnutrition and prognosis in patients with ACS and CKD has not been reported, and the prevalence of malnutrition in patients with ACS and CKD remained unknown as well.

The current guidelines recommend using the GRACE score to predict mortality and MACEs for ACS patients (6). Data from GRACE indicated that major events of patients with dialysis were largely underestimated when using the GRACE risk score (7). Hence, for patients with ACS and CKD, evaluating the incremental effect of nutrition scores in combination with the GRACE score is of great clinical significance.

In this study, we aimed to explore the prevalence and prognostic value of malnutrition in patients with ACS and CKD using different malnutrition screen tools.

## 2. Methods

### 2.1. Study population

**Figure 1:**
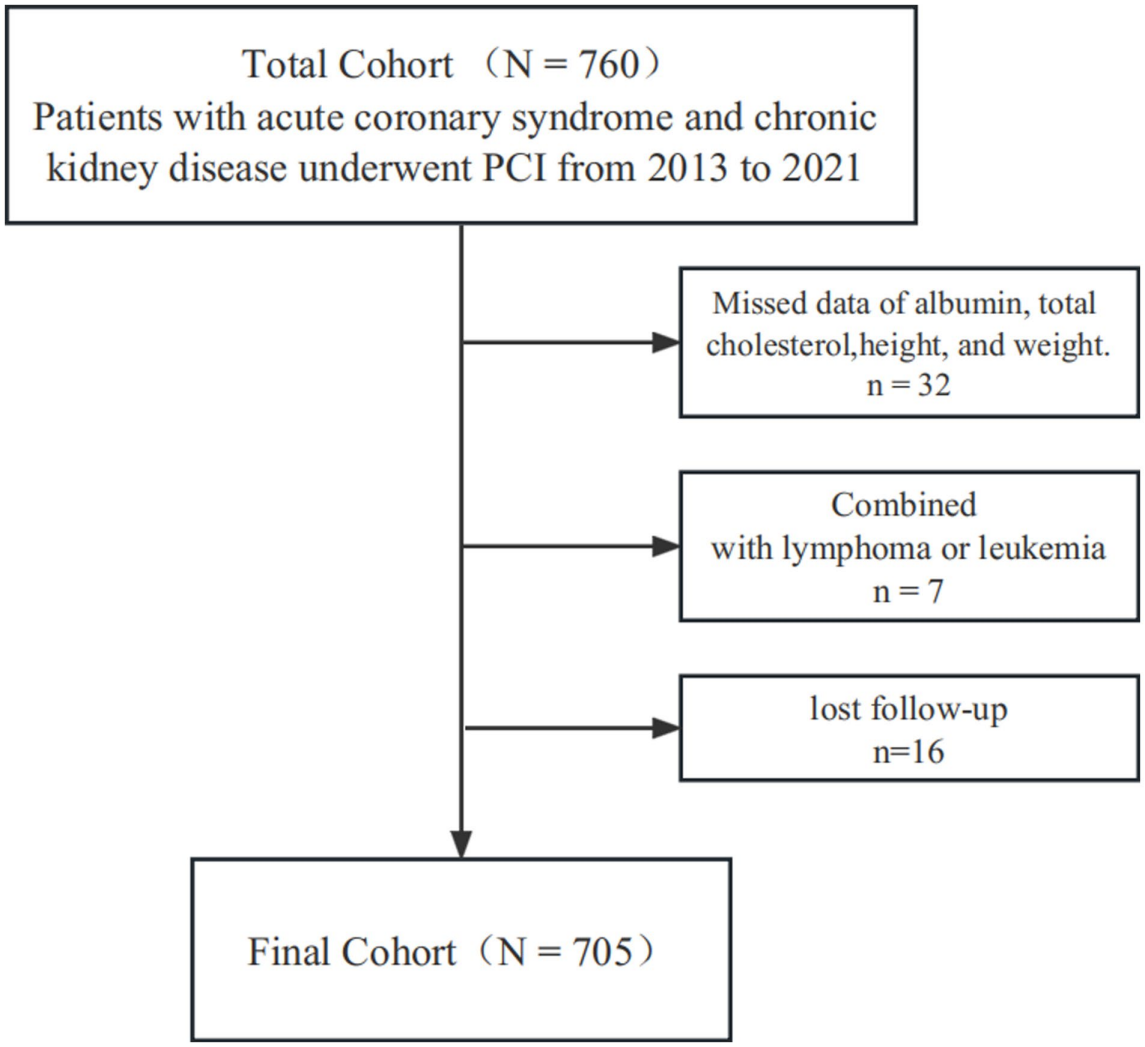
Flow chart of the study population.

A total of 760 ACS patients were diagnosed as having CKD and underwent PCI, among them 7 were combined with lymphoma or leukemia, 32 showed missing data of ALB, total cholesterol, height, and weight, 16 lost to follow-up, and eventually 705 were included in this retrospective cohort study (Figure 1). This study was carried out in the First Affiliated Hospital of Wenzhou Medical University between February 2013 and August 2021. The local ethics committee approved the study protocol.

### 2.2. Definition of clinical diseases

According to the current guidelines (18), ACS was comprised of ST-segment elevation myocardial infarction (STEMI) and non–ST-segment elevation acute coronary syndrome (NSTE-ACS). NSTE-ACS includes unstable angina and non–ST-segment elevation myocardial infarction (NSTEMI). STEMI was defined as chest discomfort or other ischaemic symptoms with new ST-segment elevations in 2 contiguous leads or new bundle branch blocks, and elevated cardiac markers. NSTEMI was defined as chest discomfort or other ischaemic symptoms with elevated cardiac markers and without ST-segment elevation on the electrocardiogram. UA was defined as newly developed or accelerated chest symptoms during 2 weeks of exertional or resting angina without elevated cardiac biomarkers. CKD was defined by an estimated glomerular filtration rate (eGFR) <60 mL/min/1.73 m^2^, including uremia. Uremia was defined as eGFR <15 mL/min per 1.73 m^2^ or receiving renal replacement therapy. The baseline SCr was measured at hospital admission. The glomerular filtration rate was calculated by serum creatinine level upon admission, according to the Chronic Kidney Disease Epidemiology Collaboration (CKD-EPI) (19). To avoid the inclusion of patients with acute kidney injury (AKI), we dynamically followed their renal function for 2 months after the inclusion of patients with baseline eGFR less than 60 mL/min per 1.73 m^2^. Those patients with recovered renal function and an eGFR greater than or equal to 60 mL/min per 1.73 m^2^ were excluded.

### 2.3. Malnutrition assessment

The nutritional status of the enrolled patients was analyzed based on the three easy-to-use scores that are effective to identify patients facing a high risk of malnutrition.

As a screening tool for the nutritional condition of hospitalized patients, the Controlling Nutritional Status (CONUT) (14) consisted of serum albumin, total cholesterol (TC) level, and lymphocyte count has been created. A score between 0 and 1 is regarded as normal; scores between 2 and 4, 5 to 8, and 9 and 12 indicate different degrees of malnutrition, including mild, moderate, and severe, respectively. The CONUT score was calculated according to the Supplementary Table S1.

The Geriatric Nutritional Risk Index (GNRI) was calculated using the following formula: 1.489*serum albumin (g/l) + 41.7*weight (kg) / ideal body weight (kg) (15). The ideal body weight was determined as follows: H − 100–[(H − 150)/4] for men and H − 100–[(H − 150)/2.5] for women, where H indicates height (cm). If the actual body weight was more than the ideal body weight, the weight-to-ideal body weight ratio was set to 1 (10, 11). These factors were obtained during the baseline assessment. Patients were categorized using the following thresholds following previous studies (10, 11): severe nutritional risk (GNRI <83.5), moderate nutritional risk (83.5 ≤ GNRI <97.5), mild nutritional risk (97.5 ≤ GNRI <100), and no nutritional risk (GNRI ≥ 100).

The Prognostic Nutritional Index (PNI) was obtained using the formula: 10*serum albumin (g/dl) + 0.005*total lymphocyte count (mm^3^) (16). A score of >38 is regarded as normal, whereas scores between 35 and 38 and < 35 indicate moderate and severe malnutrition, respectively. It should be noted that the PNI lacks a mild category.

Venous blood samples (including total cholesterol, total lymphocyte count, and serum albumin) were taken following an overnight fast at admission. To prevent variations due to measurement scale or location between laboratories, all the measurements were examined and standardized in one central laboratory.

### 2.4. Outcomes and follow-up

The primary outcome was all-cause mortality. The secondary outcome was major adverse cardiovascular events (MACEs) consists of cardiovascular death, re-infarction, or ischemic stroke. Cardiovascular death was defined as any death from cardiovascular causes. Re-infarction was defined as an recurrent elevation of cardiac enzymes greater than the upper limit of the normal range with at least one of the following: (1) recurrent ischemic symptoms, (2) electrocardiograph changes implicating ischemia, (3) development of pathological Q waves, and (4) coronary thrombus proved by angiography. Ischemic stroke was defined as ischemic lesion demonstrated by the evidence of neurological dysfunction or clinically documented lesions on imaging. Outcome information was acquired from medical records or by telephone follow-up at 1 month and then every 6 months after discharge. All patients were followed up for at least 1 month unless they died within 1 month. Follow-up was continued from the date of admission to the date of death. If there were no endpoints happened, time at the last medical encounter in primary or secondary care was taken. The median follow-up time was 31 months (inter-quartile range: 17 to 53 months). The rate of lost follow-up was 2.1%.

### 2.5. Statistical analysis

Continuous variables were expressed as the mean ± standard deviation if normally distributed or otherwise median with interquartile range (25th to 75th percentiles). Categorical variables were presented as *n* (%). The groups were compared using the chi-square test, the student t-test, and the Mann–Whitney U test for categorical data, normally distributed continuous data, and skewed data, respectively. In order to determine the relationships between nutritional indices, c-reactive protein (CRP), and eGFR, Spearman (skewed variables) correlation analysis was employed. Venn diagram was used to show how the three nutritional indexes were related to the status of malnutrition.

To show the distribution of events over time, Kaplan–Meier curves were created. Log-rank test was then used to compare groups with various nutritional statuses, followed by performing Cox proportional hazards regression analysis. Variables associated with known poor prognosis, clinical plausibility, or *p* value less than 0.05 in the univariable Cox regression analyzes were chosen for the adjusted model. The following variables were selected as covariates for the adjusted model in the present research: age, sex, BMI, hypertension, diabetes, hyperlipidemia, prior myocardial infarction, prior PCI or CABG, type of ACS, Killip class ≥ II, serum creatinine, left ventricular eject fraction (LVEF) < 40%, multivessel disease, medications at discharge including beta-blockers, ACEI/ARBs and statins, and GRACE risk score. The possible nonlinear relationships between malnutrition scoring systems and outcomes were further analyzed on a continuous scale with restricted cubic spline (RCS) curves after adjusting the variables described above, with 3 nodes at the fixed percentiles of 25, 50, and 75% of the distribution of malnutrition scores.

To assess the discrimination and reclassification performance of malnutrition scores, we combined the three scores with the GRACE score and calculated the C-statistic, continuous net reclassification improvement (NRI), and integrated discrimination improvement (IDI). The C-statistics were compared by using the Delong test (20). The GRACE score was first established for predicting 6-month mortality (6), which is derived from several clinical factors (age, heart rate, systolic blood pressure, serum creatinine, congestive heart failure, in-hospital percutaneous coronary intervention, in-hospital coronary aortic bypass grafting, history of MI, ST-segment depression, and elevated cardiac enzyme/marker levels). The web-based calculator was applied to calculate the mortality and MACEs at 6 months (6). A two-sided value of p of 0.05 was considered as statistically significant. R 4. 0. 3 and SPSS 25. 0 were used for all the analyzes.

## 3. Results

### 3.1. Baseline characteristics of the study population

Among the 705 participants with ACS and CKD in this study, 442 (62.7%) had STEMI and 263 (37.3%) had NSTE-ACS. 528 (74.89%) of the patients were male, the median BMI was 23.64 kg/m^2^, and the median age was 73 years old. Most of them were combined with hypertension (77.45%) and about one-half of the patients had hyperlipidemia (51.63%). There are 67 dialysis patients, accounting for 9.5% of the total number. Table 1 provides more details regarding the baseline characteristics.

**Table 1:** Baseline characteristics of the study population.

Variables	
*Demographic data*	
Age (years)	73 (65,80)
Male	528 (74.89%)
Height (cm)	165 (158,168)
Weight (kg)	64 (56,69)
BMI (kg/m^2^)	23.64 (21.3,25.34)
*Cardiovascular risk factors and prior procedural*	
Hypertension	546 (77.45%)
Diabetes	279 (39.57%)
Hyperlipidemia	364 (51.63%)
Dialysis	67 (9.50%)
Prior myocardial infarction	14 (1.99%)
Prior PCI	41 (5.82%)
Prior CABG	7 (0.99%)
Smoking	262 (37.16%)
*ACS presentation*	
Type of ACS	
NSTE-ACS	263 (37.3%)
STEMI	442 (62.7%)
Killip class > = II	380 (53.9%)
Laboratory data	
WBC (x10^9^/L)	9.71 (7.53,12.62)
Hb (g/L)	118 (101,133)
Platelet (x10^9^/L)	202 (165,252)
Lymphocyte (x10^9^/L)	1.29 (0.92,1.7)
Creatinine (mg/dL)	1.5 (1.28,2.17)
eGFR (mL/min/1.73m^2^)	43.54 (26.18,52.92)
TC (mg/dL)	174.02 (144.76,206.74)
Albumin (g/L)	34.4 (31.3,37.2)
CRP (mg/L)	24.5 (8.7,58.7)
FBG (mmol/l)	6.8 (5.5,9.2)
*Echocardiographic and angiographic data*	
LVEF <40%	159 (22.55%)
Multivessel disease	309 (43.83%)
LAD stenosis > = 50%	577 (81.84%)
LCX stenosis > = 50%	457 (64.82%)
RCA stenosis > = 50%	515 (73.05%)
Calcified lesions	102 (14.47%)
Thrombosis	121 (17.16%)
*Medical therapy*	
DAPT	698 (99.01%)
Beta blocker	367 (52.06%)
ACEI / ARB	211 (29.93%)
Statin	638 (90.5%)
*GRACE risk score*	135 (120,152)
*Nutrition status*	
CONUT	
Normal nutrition	137 (19.43%)
Mild malnutrition	343 (48.65%)
Moderate malnutrition	206 (29.22%)
Severe malnutrition	19 (2.7%)
PNI	
Normal nutrition	495 (70.21%)
Moderate malnutrition	104 (14.75%)
Severe malnutrition	106 (15.04%)
GNRI	
Normal nutrition	72 (10.21%)
Mild malnutrition	66 (9.36%)
Moderate malnutrition	470 (66.67%)
Severe malnutrition	97 (13.76%)

BMI, body mass index; PCI, percutaneous coronary intervention; CABG, coronary artery bypass graft; ACS, acute coronary syndrome; NSTE-ACS, non–ST-segment elevation acute coronary syndrome; STEMI, ST-segment elevation myocardial infarction; Hb, hemoglobin; eGFR, estimated glomerular filtration rate; TC, total cholesterol; CRP, c-reactive protein; FBG, fasting blood glucose; LVEF, left ventricular eject fraction; LAD, left anterior descending coronary; LCX, left circumflex artery; RCA, right coronary artery; DAPT, dual antiplatelet therapy; ACEI, angiotensin-converting enzyme inhibitor; ARB, angiotensin II receptor blocker; GRACE, Global Registry of Acute Coronary Events; CONUT, controlling nutritional status; PNI, prognostic nutritional index; GNRI, geriatric nutritional risk index.

Patients with malnutrition determined by any of the three malnutrition scores were older and were more likely to be female with lower BMI, higher CRP level, and higher GRACE risk scores than those with normal nutritional status. They also had worse clinical presentations, worse cardiac and renal functions, and were less likely to receive statins at discharge (Supplementary Tables S2–S4).

### 3.2. Prevalence of malnutrition and the population distribution

**Figure 2:**
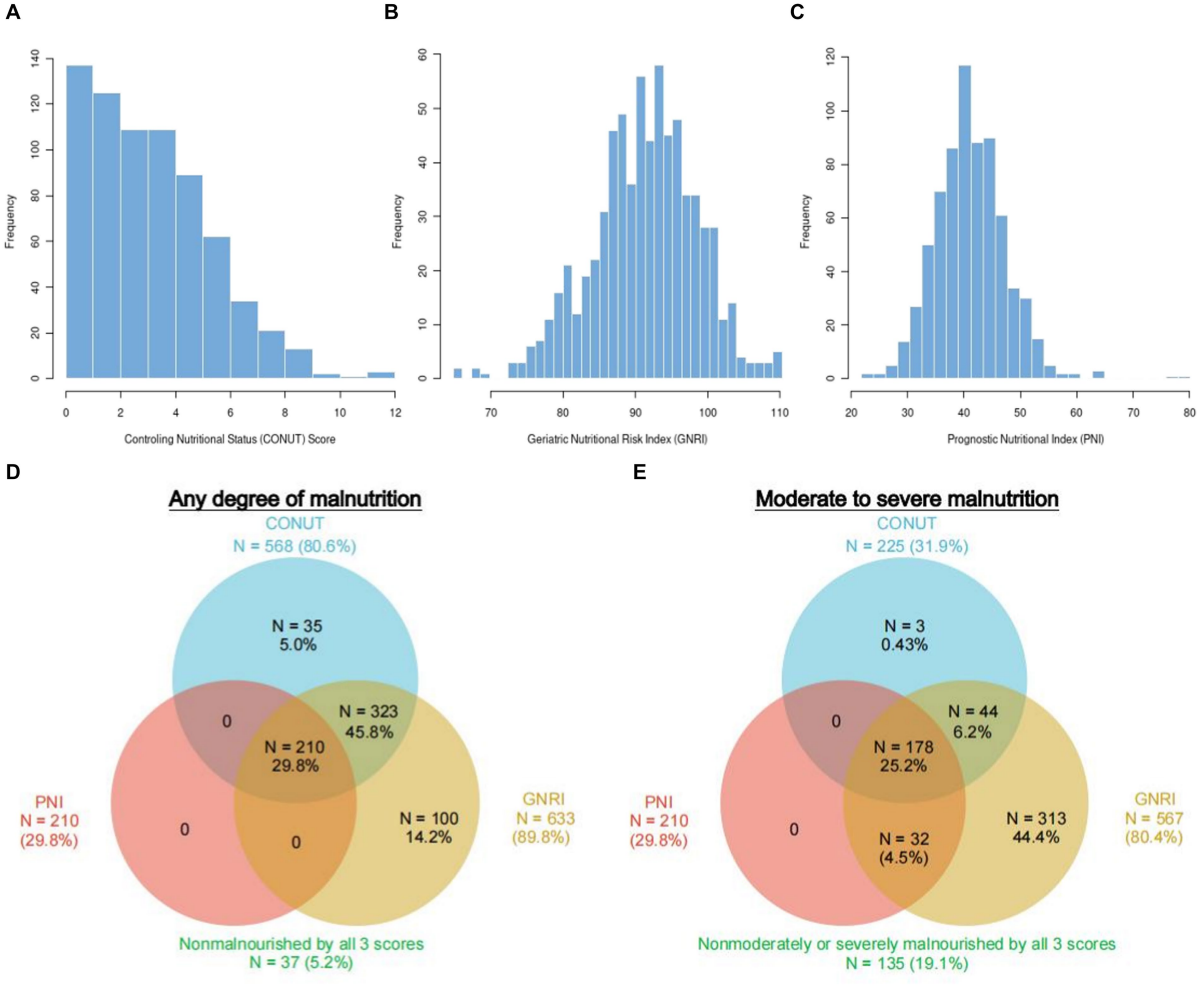
Population distribution and prevalence of malnutrition according to three different scoring systems. Histograms show the population distribution of CONUT **(A)**, GNRI **(B)**, and PNI **(C)**. The numbers reported outside each circle indicate the cumulative frequency of malnutrition. Any degree **(D)** vs. moderate–severe **(E)** according to each malnutrition score. The overlapping area of the circles reflects the frequency with which the diagnosis of malnutrition with 1 score overlaps with the others. CONUT, Controlling Nutritional Status score; GNRI, Geriatric Nutritional Risk Index; PNI, Prognostic Nutritional Index.

**Figure 3:**
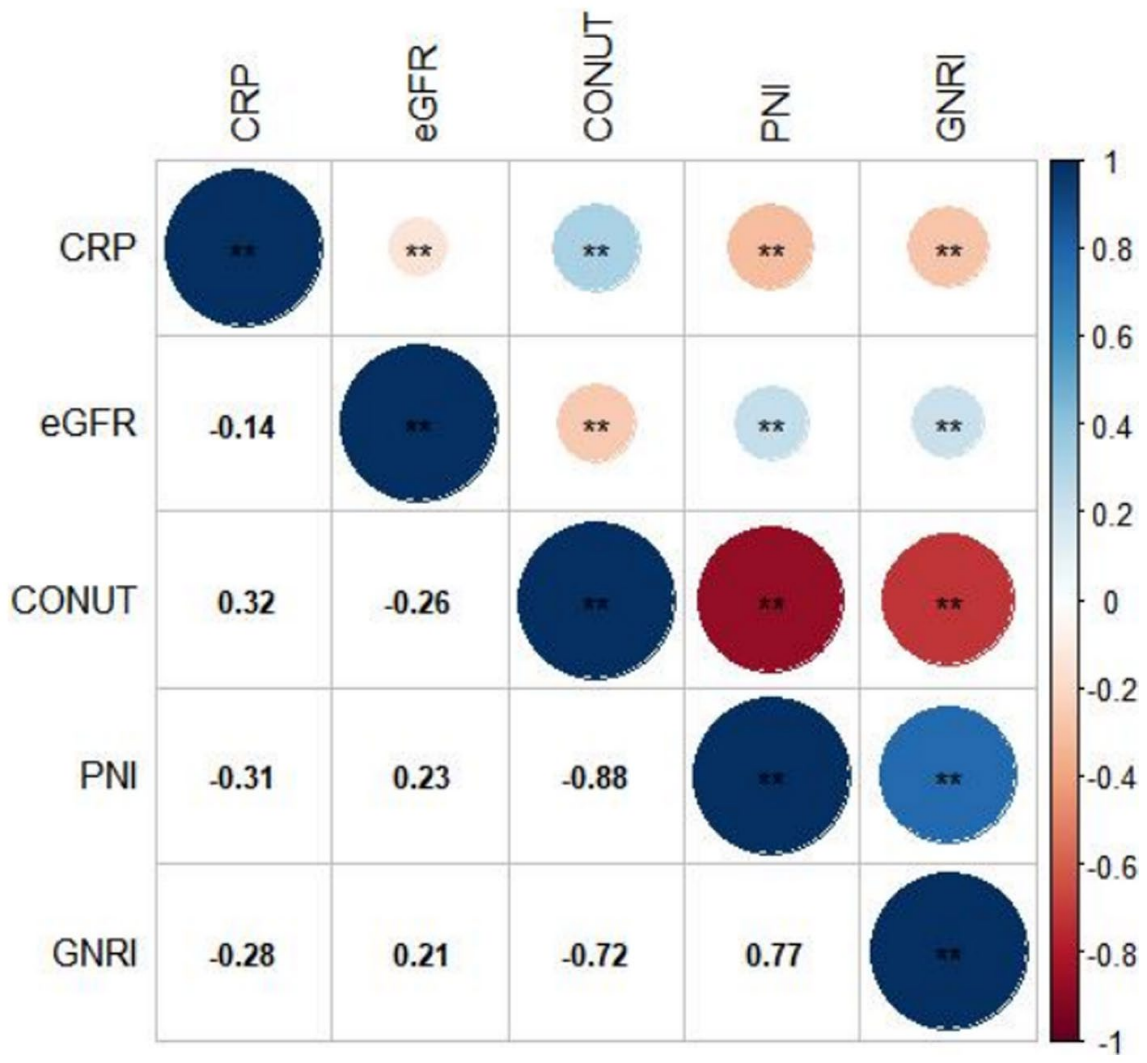
Correlation between different malnutrition indexes, CRP, and eGFR. CRP, c-reactive protein; eGFR, estimated glomerular filtration rate; CONUT, Controlling Nutritional Status score; GNRI, Geriatric Nutritional Risk Index; PNI, Prognostic Nutritional Index.

Figures 2A–C display the distributions of COUNT, GNRI, and PNI scores. Malnutrition prevalence varied from 29.8% with the PNI, to 80.6% with the CONUT, and to 89.8% with the GNRI score, as shown in Figure 2D. In addition, the CONUT and GNRI scores revealed that 225 (31.9%) and 567 (80.4%) of the patients had moderate to severe malnutrition, as shown in Figure 2E. The correlation coefficients were 0.77 between GNRI and PNI, −0.72 between GNRI and CONUT, and − 0.88 between PNI and CONUT, all with a *p* value <0.01, as shown in Figure 3. Although the malnutrition scores were correlated with each other, only 210 (29.8%) participants showed different degrees of malnutrition, and 178 (25.2%) participants showed moderate to severe malnutrition by all three nutritional scores, and only 37 (5.2%) individuals were not malnourished as calculated by any score. Furthermore, a significant correlation between CRP and the three scores was discovered (CONUT: *r* = 0.32; PNI: *r* = −0.31; GNRI: *r* = −0.28, all *p* < 0.01, Figure 3).

### 3.3. Malnutrition and clinical outcomes

During a median follow-up of 31 months (inter-quartile range: 17 to 53 months), all-cause mortality occurred to 153 (21.7%) patients and MACEs occurred to 165 (23.4%) patients. The percentage (and number) of each cardiovascular event was as follows: 12.5% (*n* = 88) cardiovascular deaths, 7.8% (*n* = 55) re-infractions, and 4.0% (*n* = 28) ischemic strokes.

**Figure 4:**
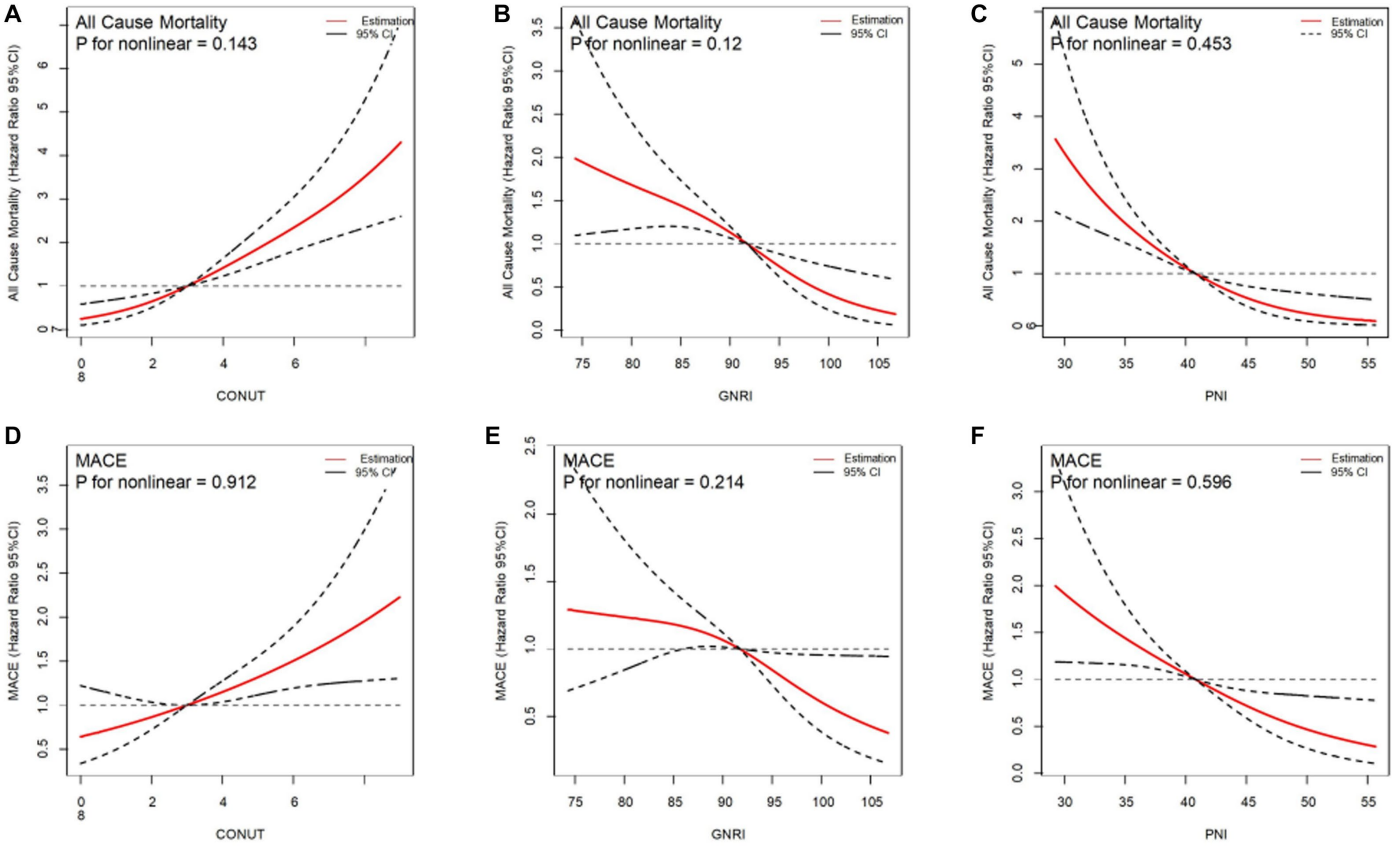
Restricted spline curves for the associations between malnutrition indexes and all-cause mortality, MACE. Red lines represent the hazard ratio, black lines represent the 95% confidence intervals. **(A)** Association between CONUT and all-cause mortality. **(B)** Association between GNRI and all-cause mortality. **(C)** Association between PNI and all-cause mortality. **(D)** Association between CONUT and MACE. **(E)** Association between GNRI and MACE. **(F)** Association beween PNI and MACE. HR (95% CI) were all adjusted according to multivariable Cox analysis.CONUT, Controlling Nutritional Status score; GNRI, Geriatric Nutritional Risk Index; PNI, Prognostic Nutritional Index; MACE, major adverse cardiovascular events.

**Figure 5:**
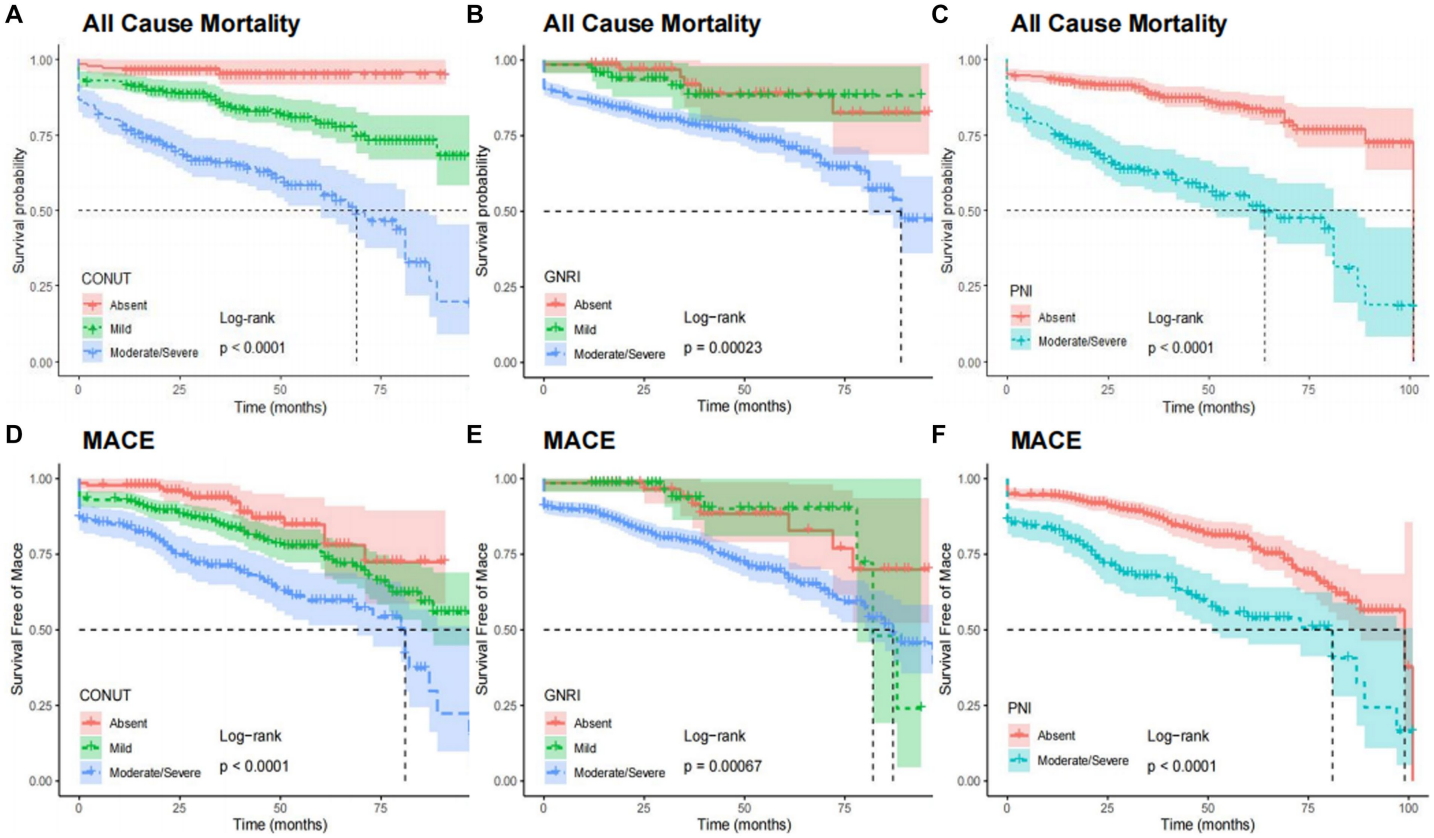
Kaplan–Meier curves for all-cause mortality and MACE by the category of each malnutrition indexes. All cause mortality by CONUT **(A)**, GNRI **(B)**, and PNI **(C)**. MACE by CONUT **(D)**, GNRI **(E)**, and PNI **(F)**. CONUT, Controlling Nutritional Status score; GNRI, geriatric nutritional risk index; PNI, prognostic nutritional index; MACE, major adverse cardiovascular events.

After adjusting the variables involved in the multivariable analysis, we used restricted cubic splines (RCS) in Figure 4 to flexibly model and illustrate the relationships of the three scoring systems with all-cause mortality and MACEs. The three indices and clinical outcomes showed no non-linear connection (all P for non-linear >0.05). Patients with any degree of malnutrition determined by the three scores had a worse prognosis for all-cause mortality and MACEs than those who did not (log-rank test, all *p* < 0.001), as demonstrated by the Kaplan–Meier analysis (Figure 5). Regardless of the malnutrition scores utilized or whether the scores were used as a continuous (Figure 4) or categorical variable (Figure 5), a worse nutritional status was linked to a higher incidence of all-cause death and MACEs.

Supplementary Table S5 displays the results of univariable Cox regression analyzes of variables in the adjusted model. The adjusted impact of the 3 malnutrition indexes on all-cause mortality and MACEs is shown in Table 2. Continuous variables of the three scores were all related to the incidence of all-cause mortality and MACEs even after adjusting (all *p* < 0.001). As for categorical variables, mild, moderate, and severe malnutrition defined by CONUT score were all independently associated with the all-cause mortality and MACEs (all *p* < 0.05), except for mild malnutrition with MACEs (HR: 1.21, 95% CI 0.67–2.17, *p* = 0.53). As for PNI, moderate and severe malnutrition were both independently associated with increased risk of mortality and MACEs when compared with normal nutrition (all *p* < 0.05). As for GNRI, only the association between severe malnutrition and all-cause mortality was statistically significant (HR: 3.16, 95% CI 1.28–7.79, *p* = 0.013).

**Table 2:** Multivariate Cox regression analyzes for all-cause mortality and MACE.

	Multivariable analysis			
	All-cause mortality		MACE	
	HR [95% CI]	*p*-value	HR [95% CI]	*p*-value
CONUT (continuous)	1.3 [1.2,1.4]	<0.001	1.15 [1.06, 1.24]	<0.001
CONUT (categorical; normal nutrition as reference)				
Mild risk	3.23 [1.24, 8.41]	0.017	1.21 [0.67, 2.17]	0.53
Moderate risk	7.23 [2.69, 19.49]	<0.001	2.08 [1.1, 3.9]	0.023
Severe risk	17.56 [5.6, 55.09]	<0.001	3.37 [1.31, 8.68]	0.012
GNRI (continuous)	0.95 [0.92, 0.97]	<0.001	0.97 [0.95, 0.99]	0.018
GNRI (categorical; normal nutrition as reference)				
Mild risk	1.4 [0.43, 4.5]	0.576	1.1 [0.39, 3.1]	0.857
Moderate risk	2.18 [0.93, 5.13]	0.075	1.75 [0.83, 3.68]	0.139
Severe risk	3.16 [1.28, 7.79]	0.013	2.05 [0.91, 4.63]	0.085
PNI (continuous)	0.89 [0.86, 0.92]	<0.001	0.93 [0.91, 0.96]	<0.001
PNI (categorical; normal nutrition as reference)				
Moderate risk	2.52 [1.62, 3.94]	<0.001	2.1 [1.4, 3.16]	<0.001
Severe risk	3.46 [2.28, 5.25]	<0.001	1.94 [1.26, 2.98]	0.003

Adjusted by age, sex, BMI, hypertension, diabetes, hyperlipidemia, prior myocardial infarction, prior PCI or CABG, type of ACS, Killip class > = II, creatinine, left ventricular eject fraction (LVEF) < 40%, multivessel disease, medications at discharge including beta-blockers, ACEI/ARBs and statins, and GRACE risk score. HR, hazard ratio; CI, confidence interval; MACE, major adverse cardiovascular event; other abbreviations as in Table 1.

**Figure 6:**
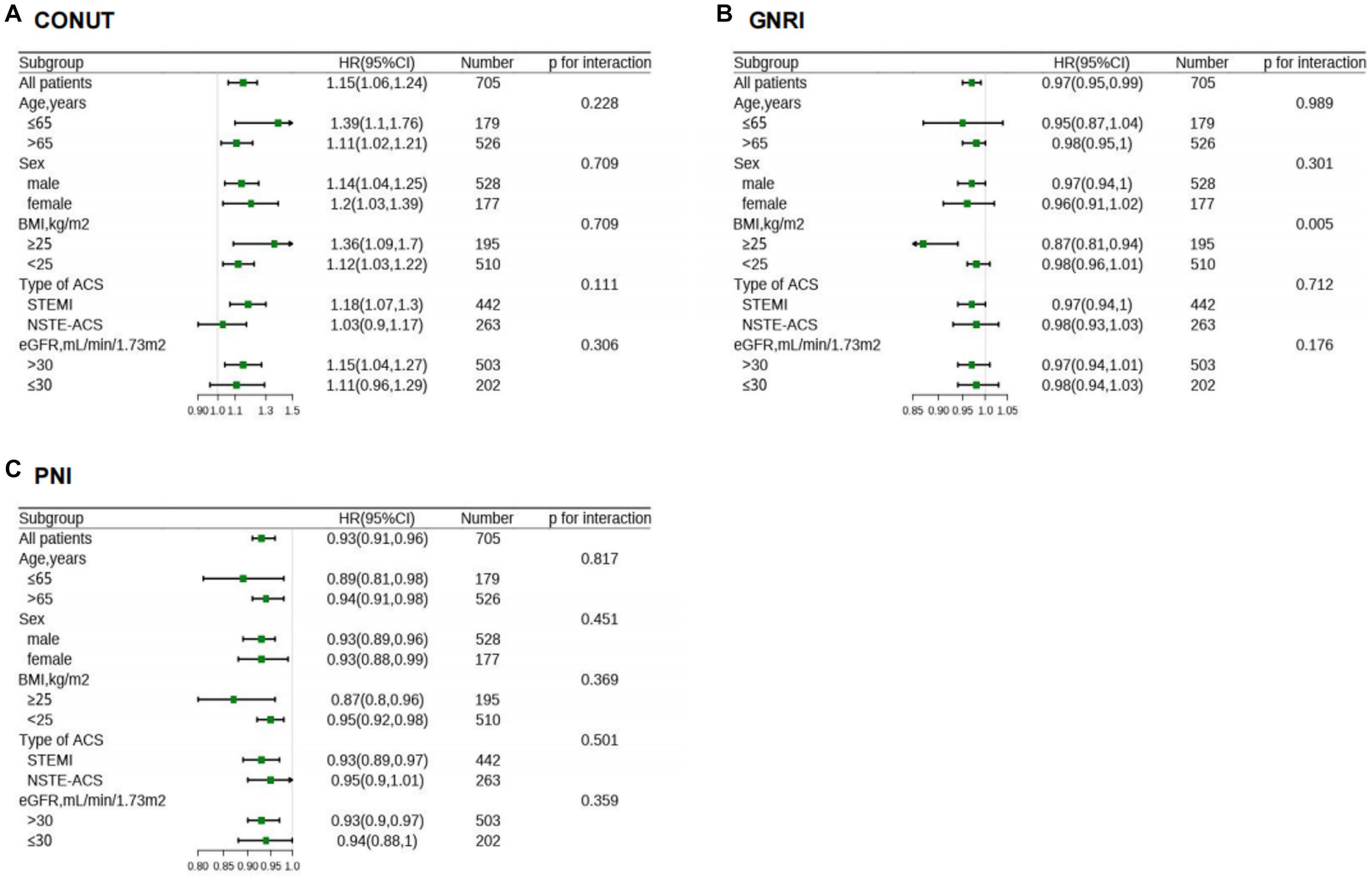
HR for MACE in different subgroups according to malnutriton scores. HR of CONUT **(A)**, GNRI **(B)**, and PNI **(C)** were adjusted by the variables as presented in Table 2, except for variable that is stratified by itself. CONUT, Controlling Nutritional Status score; GNRI, geriatric nutritional risk index; PNI, prognostic nutritional index; MACE, major adverse cardiovascular events; HR, hazard ratios; BMI, body mass index; ACS, acute coronary syndrome; NSTE-ACS, non–ST-segment elevation acute coronary syndrome; STEMI, ST-segment elevation myocardial infarction; eGFR, estimated glomerular filtration rate.

Moreover, the association between malnutrition degrees and each cardiovascular outcomes is shown in Supplementary Table S6. Subgroup analysis revealed that continuous variables of the three scores had a relatively consistent risk of MACEs across different subgroups (Age, Sex, BMI, Type of ACS, and eGFR). Also, significant interaction (P for interaction = 0.005) was observed between GNRI and BMI (Figure 6).

### 3.4. Comparative analysis and incremental value of the three nutritional scores

In Table 3, we compared the results of the three scoring systems in predicting all-cause mortality and MACEs at medium follow-up time (31 months). The discrimination index values showed that the CONUT score and PNI outperformed the GNRI for predicting all-cause mortality. For MACEs risk prediction, only the PNI was found to be significantly better than the GNRI in reclassification ability (NRI: 0.217, *p* = 0.02; IDI: 0.024, *p* = 0.02). However, the CONUT showed a higher sensitivity than the PNI, which had was of a higher specificity than the CONUT for both outcomes (all-cause death and MACEs).

**Table 3:** Comparative analysis of malnutrition scores for predicting all-cause mortality and MACE at medium follow-up time.

All-cause mortality			
Discrimination Ability	CONUT	GNRI	PNI
C-statistic (95%CI)	0.735 (0.688–0.782)	0.673 (0.623–0.724)	0.743 (0.697–0.790)
Sensitivity	0.758	0.436	0.653
1-Specificity	0.415	0.175	0.275

All-cause mortality						
Comparison	CONUT *vs* GNRI		CONUT *vs* PNI		PNI *vs* GNRI	
	Difference	*p* value	Difference	*p* value	Difference	*p* value
C-statistic	0.062	0.001	0.008	0.514	0.070	<0.001
NRI	0.169	0.007	0.06	0.445	0.346	<0.001
IDI	0.059	<0.001	0.006	0.578	0.065	<0.001

MACE			
Discrimination ability	CONUT	GNRI	PNI
C-statistic (95%CI)	0.675 (0.623–0.727)	0.657 (0.606–0.709)	0.690 (0.639–0.742)
Sensitivity	0.856	0.429	0.648
1-Specificity	0.586	0.189	0.342

MACE						
Comparison	CONUT *vs* GNRI		CONUT *vs* PNI		PNI *vs* GNRI	
	Difference	*p* value	Difference	*p* value	Difference	*p* value
C-statistic	0.018	0.373	0.015	0.274	0.033	0.071
NRI	0.07	0.299	0.106	0.193	0.217	0.02
IDI	0.012	0.266	0.011	0.159	0.024	0.02

NRI, net reclassification improvement; IDI, integrated discrimination improvement; other abbreviations as in Tables 1, 2.

Each of the three measures provided strong predictive value on the GRACE risk score for both 6-month all-cause mortality and MACEs risk prediction. The PNI showed the greatest additional value as it improved the C-statistics from 0.696 to 0.773 and from 0.653 to 0.710 for predicting mortality and MACEs, respectively. More detailed data about the incremental value are presented in Table 4.

**Table 4:** Model performance after the addition of malnutrition indexes to the GRACE Risk score for predicting 6-month all-cause mortality and MACEs.

Model	C-statistics	*p* value	NRI	*p* value	IDI	*p* value
All-cause mortality						
GRACE	0.696	ref	ref		ref	
GRACE+CONUT	0.772	<0.001	0.264	<0.001	0.077	<0.001
GRACE+GNRI	0.735	0.031	0.187	<0.001	0.044	<0.001
GRACE+PNI	0.773	<0.001	0.269	<0.001	0.092	<0.001
MACE						
GRACE	0.653	ref	ref		ref	
GRACE+CONUT	0.701	0.024	0.204	<0.001	0.034	<0.001
GRACE+GNRI	0.694	0.034	0.203	<0.001	0.031	<0.001
GRACE+PNI	0.710	0.019	0.235	<0.001	0.047	<0.001

Abbreviations as in Tables 1–3.

## 4. Discussion

To the best of our knowledge, this is the first study to report the prevalence and prognostic value of malnutrition among patients with ACS and CKD. Based on the three different malnutrition scores, the predicted prevalence of any degree malnutrition in patients with ACS and CKD varied from 29.8 to 89.8%. Depending on the scores used, the prevalence of moderate to severe malnutrition ranged from 29.8 to 80.4%. Even after adjusting age, sex, the GRACE score and other potential risk factors, malnutrition was still associated with unfavorable outcomes as predicted by any of these scores. Similar results were observed in subgroup analysis and the association between GNRI and MACEs was more pronounced in patients with BMI ≥ 25 kg/m^2^. The addition of nutrition scores to the GRACE significantly enhanced the ability to predict all-cause mortality and MACEs. Accordingly, the current findings revealed that malnutrition were prevalent in patients with ACS and CKD, and that malnutrition was an independent risk factor for all-cause mortality and MACEs regardless of the screening tools used. The addition of malnutrition scores to the existing risk score could help in better identification of high-risk patients with ACS and CKD.

Though some studies have demonstrated the benefits of invasive management of ACS in patients with CKD (21, 22), others indicated that the benefits of it in patients with impaired renal function are in fact limited (23). The management of ACS patients with CKD still remains controversial. The term “cardiorenal syndrome” describes the intricate connections between cardiac and renal disorders (24). There are numerous interfaces where cardiovascular and renal illnesses coexist. Malnutrition is one of the pathophysiologic mechanisms underlying the combined dysfunction of heart and kidneys (17). However, a large number of studies focusing on cardiovascular disease excluded patients with kidney disease, resulting in a discrepancy between clinical reality and evidentiary base, especially for patients with ACS and CKD (4). Therefore, it is necessary to investigate malnutrition in patients with ACS and CKD.

Significant correlations among the three different scores were found (Figure 3), but the prevalence of malnutrition varied depending on the nutritional screening tools. A total of 668 (94.6%) patients were diagnosed as having different degreed of malnutrition by at least one of the nutritional scores, indicating that malnutrition was prevalent in patients with ACS and CKD. The prevalence of malnutrition in patients with ACS and CKD has not been adequately studied. A previous study (25) reported that 58.5% of the participants were at moderate to severe risk of malnutrition using the GNRI score to assess a cohort of 58 patients with ACS and hemodialysis. Another study included patients with CKD detected a malnutrition rate of 94.3% among 53 patients combined with coronary artery disease (26). Our presented study demonstrated the prevalence of malnutrition in patients with ACS and CKD in a relatively larger cohort and evaluated the performance of three scores in screening malnutrition.

We discovered that even after adjusting demographic and clinical risk factors, malnutrition was still linked to all-cause mortality and MACEs. A retrospective cohort included patients with coronary heart disease (27), showed that malnutrition defined by the CONUT score was associated with all-cause mortality, and that the association was also observed among CKD patients. In addition, research from Tokyo (28) also showed that malnutrition was a strong risk factor for MACEs among patients with stable coronary heart disease, and that the association remained even stratified by CKD. However, the findings of the two researches are not in line with a past report (25), in which malnutrition was not linked to in-hospital and long-term mortality in patients with ACS and hemodialysis after adjustment. Due to its relatively small sample size (*n* = 58) and less representative CKD population, the last study (25) should be treated with caution.

Malnutrition is a complex pathological condition. Nutritional status may be a proxy indication of inflammation, which could explain the association between unfavorable prognosis and malnutrition in ACS and CKD patients (29). The present study observed a significant correlation between CRP levels and malnutrition (Figure 2). ACS is the result of atherosclerotic plaque rupture caused by chronic inflammatory response (30). As two proven non-traditional risk factors for people with CKD, inflammation and malnutrition could interact with each other and promote the development of atherosclerosis, resulting in cardiovascular events or death (9, 31, 32). This progress becomes even worse with the decline in renal function. Worse malnutrition is associated with elevated levels of inflammation, which accelerates the progress of atherosclerosis. The relationship between these three conditions has been currently defined as Malnutrition-Inflammation-Atherosclerosis Syndrome (33). Moreover, malnutrition is a complex disorder that includes decreased protein reserves and caloric collapse, both of which could impair immune system. Several chronic diseases emerge and develop as a result of the collapse of immune system. Reduced nutritional intake, poor nutrient absorption, and disorders of neurohormonal and promote the progression of malnutrition (17). Consequently, a vicious cycle could be resulted from a positive feedback loop between immune defense, malnutrition, inflammation, and unfavorable consequences.

Standards for malnutrition screening tools have not been established yet. In our study, the PNI demonstrated the strongest predictive performance for both all-cause mortality and MACEs, while the predictive ability of GNRI was the lowest (Tables 3, 4). The CONUT contained serum albumin, total cholesterol levels, and total lymphocyte count for assessing nutritional status. The PNI consisted of albumin and lymphocyte count, and the GNRI considered serum albumin levels, body weight, and height (14–16). The common element among the three nutritional indices, that is, albumin, reflected both systemic inflammation and nutritional status (34). Even among healthy people, hypoalbuminemia was linked to death regardless of the underlying illness (35). The immunological regulatory response could be represented in lymphocytes. Lymphocyte count decline is thought to be a result of acute stress or gradual depletion of bodily reserves, and it is linked to unfavorable prognosis of those with kidney disease and cardiovascular disease (36, 37). Recent studies showed that patients with ACS or CKD who had lower BMI were more likely to die and experience cardiovascular events (11–13, 38), which was consistent with our finding in the unadjusted Cox regression analyzes that BMI was negatively associated with all-cause mortality (Supplementary Table S4). However, it is essential to emphasize that body weight is influenced by edema and urine output, which may increase the measured weight of participants with CKD than their actual weight and therefore affect the predictive ability of the GNRI. In addition, GNRI was found to interacted with BMI (p for interaction = 0.005) regarding MACEs, which was due to the fact that GNRI was created to address the challenges associated with determining appropriate weight, and that both GNRI and BMI estimates consider height and weight (15). Our research indicated that PNI outperformed the COUNT score in terms of predicting all-cause mortality and MACEs. We speculated that this was because certain factors, such as dietary choices, drug usage, alcohol use, smoking, and lifestyle choices, can affect total cholesterol (TC), a component of the CONUT score. Moreover, serum albumin concentrations, total lymphocyte count, and TC were all categorical variables in the COUNT score, making it a rough measure to capture the additional predictive information. Based on our findings, we recommended the application of the PNI for the first time, which only requires two laboratory indices and was relatively simple to calculate—even without specialized automatic calculators.

The GRACE score is recommended in the current guidelines for risk stratification and prognostic evaluation in ACS patients (6, 39). The components of the GRACE score include serum creatinine level and cardiac biomarker level, which could be elevated with the decline in renal function (40). Hence, the prognostic impact of these variables would be reduced among patients with CKD. Moreover, the GRACE score is generated using unselected and generalizable patients (6), which may limit its application among specific populations such as those with CKD (7). Our findings indicated that the GRACE score could be greatly improved by including the malnutrition score for a better prediction of MACEs and all-cause mortality. Thus, we highlighted the necessary for physicians to incorporate the detection of malnutrition into their routine practice to enhance risk stratification and guide the following secondary prevention interventions.

How to prevent and treat malnutrition? Various strategies have been developed including oral nutritional supplements, dietary interventions, food/liquid fortification or enrichment, and certain public health measures (41). Certain diets are associated with the risk of malnutrition and cardiovascular complications. The ketogenic diet (KD) is protective for malnutrition risk and cardiovascular complications like diabetes and obesity, by altering the homeostasis of metabolites and regulating the level of glucose sugar and insulin (42–44). In contrast, the high sugar diet mediates insulin resistance, reduced fatty acid oxidation, and dyslipidemia (45), and is associated with an increased risk of sarcopenia and major cardiovascular events, both among participants with preexisting cardiovascular disease and those without such disease (46, 47). Government departments should implement public health measures like providing education/information on healthy diets, implementing nutrition labeling in processed food, exploring oil of high antioxidant activity, and encouraging the cultivation of plants with high protein content to promote protein intake in patients with malnutrition (48–50). Although dietary supplements could treat malnutrition, they should be considered as part of the whole medication. Elderly people, especially patients with cardiovascular comorbidities, have a high risk of inappropriate and excessive medication (51). Excess medication interacts with the cytochrome P450 enzyme system of the liver and affects absorption (52). Cardiovascular comorbidities and malnutrition can lower serum albumin levels, and renal function can decline with aging, which in turn increases the toxicity, alter the pharmacodynamics of drugs, and eventually lead to prolonged admission, greater hospital costs, and high mortality for elderly patients (52, 53). Medications such as glucagon-like peptide 1 (GLP-1) agonists, glucose-dependent insulinotropic polypeptide (GIP) agonists, and dual GLP-1/GIP agonists could benefit patients with diabetes, obesity, and cardiovascular disease (54). In the future, novel medications that have pleiotropic effects are warranted to reduce the number of administered drugs.

The current study has several limitations to be noted. Firstly, the single-centered and retrospective design of this study has certain inherent drawbacks including differences in medical level in different periods. However, it included patients treated in actual clinical settings in accordance with standardized institutional protocols, thereby eliminating many possible confounding factors from selection bias and heterogeneous clinical practice. Secondly, Malnutrition data was only collected at the baseline, making it difficult to theoretically explain potential changes in each parameter over time, but it was still a reliable approach to assessing long-term effects of malnutrition according to previous reports (5, 10, 11). Thirdly, we did not compare indicators with more complex comprehensive nutritional assessments, such as GLIM criteria and NRS-2002. Lastly, further studies are encouraged to ascertain whether the preoperative correction of malnutrition and initial nutritional intervention could enhance postoperative outcomes.

Conclusively, the current findings revealed that malnutrition scores were applicable to screen for malnutrition in patients with ACS and CKD, and that malnutrition was an independent risk factor for all-cause mortality and MACEs regardless of the screening tools used. Identifying malnutrition and those who might benefit from nutritional supplement is significant for a favorable prognosis of patients with ACS and CKD.

## Data availability statement

The raw data supporting the conclusion of this article will be made available by the authors, without undue reservation.

## Ethics statement

The First Affiliated Hospital of the Ethics Committee in Clinical Research of Wenzhou Medical University examined and approved the present research involving human subjects. The participants provided written informed consent before being enrolled in this trial. Any identifying photographs or data included in this manuscript were published with the consent of the informed individuals.

## Author contributions

WN, KG, and HZ conceived and designed the study. WN, SS, and LC acquired the data and drafted the manuscript. YZ, FZ, JX, and KL analyzed the data. ZG, CC, and HZ contributed to the interpretation of the results and critical revision of the manuscript for important intellectual content. HZ had primary responsibility for final content. All authors contributed to the article and approved the submitted version.

## Funding

The study was funded by the National Natural Science Foundation of China (81800048 and 81873468) and the Science and Technology Planning Project of Wenzhou Science & Technology Bureau of Zhejiang Province of China (Y20160118).

## Conflict of interest

The authors declare that the research was conducted in the absence of any commercial or financial relationships that could be construed as a potential conflict of interest.

## Publisher’s note

All claims expressed in this article are solely those of the authors and do not necessarily represent those of their affiliated organizations, or those of the publisher, the editors and the reviewers. Any product that may be evaluated in this article, or claim that may be made by its manufacturer, is not guaranteed or endorsed by the publisher.

## References

[1] Go AS Chertow GM Fan D McCulloch CE Hsu CY. Chronic kidney disease and the risks of death, cardiovascular events, and hospitalization. N Engl J Med. (2004) 351:1296–305. doi: 10.1056/NEJMoa041031 15385656

[2] Fox CS Muntner P Chen AY Alexander KP Roe MT Cannon CP. Use of evidence-based therapies in short-term outcomes of ST-segment elevation myocardial infarction and non-ST-segment elevation myocardial infarction in patients with chronic kidney disease. Circulation. (2010) 121:357–65. doi: 10.1161/circulationaha.109.865352, PMID: 20065168 PMC2874063

[3] Sarnak MJ Amann K Bangalore S Cavalcante JL Charytan DM Craig JC. Chronic kidney disease and coronary artery disease: JACC state-of-the-art review. J Am Coll Cardiol. (2019) 74:1823–38. doi: 10.1016/j.jacc.2019.08.1017 31582143

[4] Konstantinidis I Nadkarni GN Yacoub R Saha A Simoes P Parikh CR. Representation of patients with kidney disease in trials of cardiovascular interventions: an updated systematic review. JAMA Intern Med. (2016) 176:121–4. doi: 10.1001/jamainternmed.2015.6102, PMID: 26619332

[5] Ishida JH Johansen KL. Exclusion of patients with kidney disease from cardiovascular trials. JAMA Intern Med. (2016) 176:124–5. doi: 10.1001/jamainternmed.2015.6403, PMID: 26618994 PMC4701578

[6] Eagle KA Lim MJ Dabbous OH Pieper KS Goldberg RJ van de Werf F. A validated prediction model for all forms of acute coronary syndrome: estimating the risk of 6-month postdischarge death in an international registry. JAMA. (2004) 291:2727–33. doi: 10.1001/jama.291.22.2727, PMID: 15187054

[7] Gurm HS Gore JM Anderson FA Jr Wyman A Fox KAA Steg PG. Comparison of acute coronary syndrome in patients receiving versus not receiving chronic dialysis (from the global registry of acute coronary events [GRACE] registry). Am J Cardiol. (2012) 109:19–25. doi: 10.1016/j.amjcard.2011.07.062, PMID: 21974963

[8] Zhang Q Qian L Liu T Ding JS Zhang X Song MM. Prevalence and prognostic value of malnutrition among elderly cancer patients using three scoring systems. Front Nutr. (2021) 8:738550. doi: 10.3389/fnut.2021.738550, PMID: 34708064 PMC8544751

[9] Zha Y Qian Q. Protein nutrition and malnutrition in CKD and ESRD. Nutrients. (2017) 9:208. doi: 10.3390/nu9030208, PMID: 28264439 PMC5372871

[10] Sze S Pellicori P Kazmi S Rigby A Cleland JGF Wong K. Prevalence and prognostic significance of malnutrition using 3 scoring systems among outpatients with heart failure: a Comparison with body mass index. JACC Heart Fail. (2018) 6:476–86. doi: 10.1016/j.jchf.2018.02.018, PMID: 29753673

[11] Raposeiras Roubín S Abu Assi E Cespón Fernandez M Barreiro Pardal C Lizancos Castro A Parada JA. Prevalence and prognostic significance of malnutrition in patients with acute coronary syndrome. J Am Coll Cardiol. (2020) 76:828–40. doi: 10.1016/j.jacc.2020.06.058, PMID: 32792081

[12] Chen X Xiong S Chen Y Cheng L Chen Q Yang S. The predictive value of different nutritional indices combined with the GRACE score in predicting the risk of long-term death in patients with acute coronary syndrome undergoing percutaneous coronary intervention. J Cardiovasc Dev Dis. (2022) 9:358. doi: 10.3390/jcdd9100358 36286310 PMC9604676

[13] Ma XT Shao QY Li QX Yang ZQ Han KN Liang J. Nutritional risk index improves the GRACE score prediction of clinical outcomes in patients with acute coronary syndrome undergoing percutaneous coronary intervention. Front Cardiovasc Med. (2021) 8:773200. doi: 10.3389/fcvm.2021.773200, PMID: 34977188 PMC8716456

[14] Ignacio de Ulíbarri J González-Madroño A de Villar NG González P González B Mancha A. CONUT: a tool for controlling nutritional status. First validation in a hospital population. Nutr Hosp. (2005) 20:38–45. PMID: 15762418

[15] Bouillanne O Morineau G Dupont C Coulombel I Vincent JP Nicolis I. Geriatric nutritional risk index: a new index for evaluating at-risk elderly medical patients. Am J Clin Nutr. (2005) 82:777–83. doi: 10.1093/ajcn/82.4.777, PMID: 16210706

[16] Buzby GP Mullen JL Matthews DC Hobbs CL Rosato EF. Prognostic nutritional index in gastrointestinal surgery. Am J Surg. (1980) 139:160–7. doi: 10.1016/0002-9610(80)90246-9 7350839

[17] Hatamizadeh P Fonarow GC Budoff MJ Darabian S Kovesdy CP Kalantar-Zadeh K. Cardiorenal syndrome: pathophysiology and potential targets for clinical management. Nat Rev Nephrol. (2013) 9:99–111. doi: 10.1038/nrneph.2012.279, PMID: 23247571

[18] Thygesen K Alpert JS Jaffe AS Chaitman BR Bax JJ Morrow DA. Fourth universal definition of myocardial infarction (2018). J Am Coll Cardiol. (2018) 72:2231–64. doi: 10.1016/j.jacc.2018.08.1038 30153967

[19] Levey AS Stevens LA Schmid CH Zhang Y(L) Castro AF III Feldman HI. A new equation to estimate glomerular filtration rate [published correction appears in Ann intern med. 2011 Sep 20;155(6):408]. Ann Intern Med. (2009) 150:604–12. doi: 10.7326/0003-4819-150-9-200905050-00006, PMID: 19414839 PMC2763564

[20] DeLong ER DeLong DM Clarke-Pearson DL. Comparing the areas under two or more correlated receiver operating characteristic curves: a nonparametric approach. Biometrics. (1988) 44:837–45. doi: 10.2307/2531595 3203132

[21] Huang HD Alam M Hamzeh I Virani S Deswal A Aguilar D. Patients with severe chronic kidney disease benefit from early revascularization after acute coronary syndrome. Int J Cardiol. (2013) 168:3741–6. doi: 10.1016/j.ijcard.2013.06.013 23845772

[22] Wong JA Goodman SG Yan RT Wald R Bagnall AJ Welsh RC. Temporal management patterns and outcomes of non-ST elevation acute coronary syndromes in patients with kidney dysfunction. Eur Heart J. (2009) 30:549–57. doi: 10.1093/eurheartj/ehp014, PMID: 19201761

[23] Szummer K Lundman P Jacobson SH Schön S Lindbäck J Stenestrand U. Influence of renal function on the effects of early revascularization in non-ST-elevation myocardial infarction: data from the Swedish web-system for enhancement and development of evidence-based Care in Heart Disease Evaluated According to recommended therapies (SWEDEHEART). Circulation. (2009) 120:851–8. doi: 10.1161/CIRCULATIONAHA.108.838169, PMID: 19704097

[24] Rangaswami J Bhalla V Blair JEA Chang TI Costa S Lentine KL. Cardiorenal syndrome: classification, pathophysiology, diagnosis, and treatment strategies: a scientific statement from the American Heart Association. Circulation. (2019) 139:e840–78. doi: 10.1161/CIR.0000000000000664, PMID: 30852913

[25] Kobayashi H Takahashi M Fukutomi M Oba Y Funayama H Kario K. The long-term prognostic factors in hemodialysis patients with acute coronary syndrome: perspectives from sarcopenia and malnutrition. Heart Vessel. (2021) 36:1275–82. doi: 10.1007/s00380-021-01815-0, PMID: 33677618

[26] Xi WZ Wu C Liang YL Wang LL Cao YH. Analysis of malnutrition factors for inpatients with chronic kidney disease. Front Nutr. (2023) 9:1002498. doi: 10.3389/fnut.2022.1002498, PMID: 36687720 PMC9852827

[27] Liu J Huang Z Huang H He Y Yu Y Chen G. Malnutrition in patients with coronary artery disease: prevalence and mortality in a 46,485 Chinese cohort study. Nutr Metab Cardiovasc Dis. (2022) 32:1186–94. doi: 10.1016/j.numecd.2021.12.023 35260308

[28] Wada H Dohi T Miyauchi K Doi S Konishi H Naito R. Prognostic impact of nutritional status assessed by the controlling nutritional status score in patients with stable coronary artery disease undergoing percutaneous coronary intervention. Clin Res Cardiol. (2017) 106:875–83. doi: 10.1007/s00392-017-1132-z, PMID: 28634674

[29] Kalantar-Zadeh K Anker SD Horwich TB Fonarow GC. Nutritional and anti-inflammatory interventions in chronic heart failure. Am J Cardiol. (2008) 101:89E–103E. doi: 10.1016/j.amjcard.2008.03.007, PMID: 18514634 PMC5500213

[30] Lehrke M Millington SC Lefterova M Cumaranatunge RG Szapary P Wilensky R. CXCL16 is a marker of inflammation, atherosclerosis, and acute coronary syndromes in humans. J Am Coll Cardiol. (2007) 49:442–9. doi: 10.1016/j.jacc.2006.09.034, PMID: 17258089

[31] Rodin R Chan CT. Determinants and prevention of coronary disease in patients with chronic kidney disease. Can J Cardiol. (2019) 35:1181–7. doi: 10.1016/j.cjca.2019.05.025 31472816

[32] Peev V Nayer A Contreras G. Dyslipidemia, malnutrition, inflammation, cardiovascular disease and mortality in chronic kidney disease. Curr Opin Lipidol. (2014) 25:54–60. doi: 10.1097/MOL.0000000000000045, PMID: 24345987

[33] Sueta D Hokimoto S Sakamoto K Akasaka T Tabata N Kaikita K. Validation of the high mortality rate of malnutrition-inflammation-atherosclerosis syndrome: -community-based observational study. Int J Cardiol. (2017) 230:97–102. doi: 10.1016/j.ijcard.2016.12.072, PMID: 28038804

[34] Soeters PB Wolfe RR Shenkin A. Hypoalbuminemia: pathogenesis and clinical significance. JPEN J Parenter Enteral Nutr. (2019) 43:181–93. doi: 10.1002/jpen.1451, PMID: 30288759 PMC7379941

[35] Franch-Arcas G. The meaning of hypoalbuminaemia in clinical practice. Clin Nutr. (2001) 20:265–9. doi: 10.1054/clnu.2001.0438, PMID: 11407875

[36] Núñez J Miñana G Bodí V Núñez E Sanchis J Husser O. Low lymphocyte count and cardiovascular diseases. Curr Med Chem. (2011) 18:3226–33. doi: 10.2174/092986711796391633 21671854

[37] Kim JU Kim M Kim S Nguyen TT Kim E Lee S. Dendritic cell dysfunction in patients with end-stage renal disease. Immune Netw. (2017) 17:152–62. doi: 10.4110/in.2017.17.3.152, PMID: 28680376 PMC5484645

[38] Ladhani M Craig JC Irving M Clayton PA Wong G. Obesity and the risk of cardiovascular and all-cause mortality in chronic kidney disease: a systematic review and meta-analysis. Nephrol Dial Transplant. (2017) 32:gfw075–449. doi: 10.1093/ndt/gfw075, PMID: 27190330

[39] Collet JP Thiele H Barbato E Barthélémy O Bauersachs J Bhatt DL. 2020 ESC guidelines for the management of acute coronary syndromes in patients presenting without persistent ST-segment elevation. Eur Heart J. (2021) 42:1289–367. doi: 10.1093/eurheartj/ehaa575 32860058

[40] Li D Jialal I Keffer J. Greater frequency of increased cardiac troponin T than increased cardiac troponin I in patients with chronic renal failure. Clin Chem. (1996) 42:114–5. doi: 10.1093/clinchem/42.1.114, PMID: 8565211

[41] Bandayrel K Wong S. Systematic literature review of randomized control trials assessing the effectiveness of nutrition interventions in community-dwelling older adults. J Nutr Educ Behav. (2011) 43:251–62. doi: 10.1016/j.jneb.2010.01.004 21371944

[42] Kumar S Behl T Sachdeva M Sehgal A Kumari S Kumar A. Implicating the effect of ketogenic diet as a preventive measure to obesity and diabetes mellitus. Life Sci. (2021) 264:118661. doi: 10.1016/j.lfs.2020.118661, PMID: 33121986

[43] Ma DC Anderson CM Rodman SN Buranasudja V McCormick ML Davis A. Ketogenic diet with concurrent chemoradiation in head and neck squamous cell carcinoma: preclinical and phase 1 trial results. Radiat Res. (2021) 196:213–24. doi: 10.1667/RADE-20-00150.1, PMID: 34087943 PMC8440425

[44] Shukla SK Gebregiworgis T Purohit V Chaika NV Gunda V Radhakrishnan P. Metabolic reprogramming induced by ketone bodies diminishes pancreatic cancer cachexia. Cancer Metab. (2014) 2:18. doi: 10.1186/2049-3002-2-18 25228990 PMC4165433

[45] Stanhope KL. Sugar consumption, metabolic disease and obesity: the state of the controversy. Crit Rev Clin Lab Sci. (2016) 53:52–67. doi: 10.3109/10408363.2015.1084990, PMID: 26376619 PMC4822166

[46] Argilés JM Campos N Lopez-Pedrosa JM Rueda R Rodriguez-Mañas L. Skeletal muscle regulates metabolism via interorgan crosstalk: roles in health and disease. J Am Med Dir Assoc. (2016) 17:789–96. doi: 10.1016/j.jamda.2016.04.019, PMID: 27324808

[47] Jenkins DJA Dehghan M Mente A Bangdiwala SI Rangarajan S Srichaikul K. Glycemic index, Glycemic load, and cardiovascular disease and mortality. N Engl J Med. (2021) 384:1312–22. doi: 10.1056/NEJMoa2007123, PMID: 33626252

[48] Gao C Xu J Liu Y Yang Y. Nutrition policy and healthy China 2030 building. Eur J Clin Nutr. (2021) 75:238–46. doi: 10.1038/s41430-020-00765-6, PMID: 33219269

[49] Copolovici D Bungau S Boscencu R Tit D Copolovici L. The fatty acids composition and antioxidant activity of walnut cold press oil. Rev Roum Chim. (2017) 2017:507–9. doi: 10.37358/RC.17.3.5489

[50] Ramya KR Tripathi K Pandey A Barpete S Gore PG Raina AP. Rediscovering the potential of multifaceted orphan legume Grasspea-a sustainable resource with high nutritional values. Front Nutr. (2022) 8:826208. doi: 10.3389/fnut.2021.826208, PMID: 35281763 PMC8906286

[51] Krustev T Milushewa P Tachkov K Mitov K Petrova G. Evaluation of potentially inappropriate medication in older patients with cardiovascular diseases-STOPP/START-based study. Front. Public Health. (2022) 10:1023171. doi: 10.3389/fpubh.2022.1023171, PMID: 36620233 PMC9813954

[52] Lavan AH Gallagher PF O’Mahony D. Methods to reduce prescribing errors in elderly patients with multimorbidity. Clin Interv Aging. (2016) 11:857–66. Published 2016 Jun 23. doi: 10.2147/CIA.S80280, PMID: 27382268 PMC4922820

[53] Khan LM. Comparative epidemiology of hospital-acquired adverse drug reactions in adults and children and their impact on cost and hospital stay--a systematic review. Eur J Clin Pharmacol. (2013) 69:1985–96. doi: 10.1007/s00228-013-1563-z 23955174

[54] Vesa CM Bungau SG. Novel molecules in diabetes mellitus, Dyslipidemia and cardiovascular disease. Int J Mol Sci. (2023) 24:4029. doi: 10.3390/ijms24044029 36835441 PMC9961468

