# Supplementary material for: Prevalence and prognostic value of malnutrition in patients with acute coronary syndrome and chronic kidney disease
Source: Front Nutr. 2023 Jul 14;10:1187672. doi: 10.3389/fnut.2023.1187672 (PMC10376694; doi:10.3389/fnut.2023.1187672)
Supplement: Supplementary file 1 [file Table_1.DOCX]

Supplement table 1. CONUT score

| **Variables** | **Malnutrition** | | | |
| --- | --- | --- | --- | --- |
|  | **Absent**  **0-1** | **Mild**  **2-4** | **Moderate**  **5-8** | **Severe**  **9-12** |
| **Albumin, g/dl (score)** | ≥3.5 (0) | 3.0-3.4 (2) | 2.5-2.9 (4) | <2.5 (6) |
| **Total cholesterol, mmol/l (score)** | ≥180 (0) | 140-199 (1) | 100-139 (2) | <100 (3) |
| **Lymphocyte count, x10^9^/l (score)** | ≥1.60 (0) | 1.20-1.59 (1) | 0.80 - 1.19 (2) | <0.80 (3) |
