# Supplementary material for: Prevalence and prognostic value of malnutrition in patients with acute coronary syndrome and chronic kidney disease
Source: Front Nutr. 2023 Jul 14;10:1187672. doi: 10.3389/fnut.2023.1187672 (PMC10376694; doi:10.3389/fnut.2023.1187672)
Supplement: Supplementary file 2 [file Table_2.DOCX]

Supplement table 2. Baseline characteristics by CONUT categories

| **Variables** | **CONUT** | | | | **P-value** |
| --- | --- | --- | --- | --- | --- |
|  | **0-1**  **Normal**  **(n=137)** | **2-4**  **Mild**  **(n=343)** | **5-8**  **Moderate**  **(n=206)** | **9-12**  **Severe**  **(n=19)** |  |
| **Age (years)** | 67 (61,77) | 73 (66,79) | 75 (67,81) | 75 (72,81) | <0.001 |
| **Male** | 113 (82.48%) | 260 (75.8%) | 138 (66.99%) | 17 (89.47%) | 0.004 |
| **Height (cm)** | 165 (159,168) | 165 (160,168) | 165 (156.25,168) | 165.3 (161.5,168.5) | 0.433 |
| **Weight (kg)** | 65 (59,70) | 63 (56,69) | 60 (55,67.88) | 64.6 (58.5,66.55) | 0.009 |
| **BMI (kg/m^2^）** | 23.88 (22.15,26.67) | 23.53 (21.58,25.17) | 23.2 (20.67,24.85) | 23.64 (21.93,24.97) | 0.002 |
| **Hypertension** | 110 (80.29%) | 274 (79.88%) | 150 (72.82%) | 12 (63.16%) | 0.088 |
| **Diabetes** | 42 (30.66%) | 138 (40.23%) | 92 (44.66%) | 7 (36.84%) | 0.075 |
| **Hyperlipidemia** | 103 (75.18%) | 186 (54.23%) | 72 (34.95%) | 3 (15.79%) | <0.001 |
| **Dialysis** | 5 (3.65%) | 29 (8.45%) | 32 (15.53%) | 1 (5.26%) | <0.001 |
| **Prior myocardial infarction** | 1 (0.73%) | 6 (1.75%) | 6 (2.91%) | 1 (5.26%) | 0.259 |
| **Prior PCI** | 7 (5.11%) | 20 (5.83%) | 14 (6.8%) | 0 (0%) | 0.646 |
| **Prior CABG** | 1 (0.73%) | 4 (1.17%) | 2 (0.97%) | 0 (0%) | 1 |
| **Smoking** | 60 (43.8%) | 133 (38.78%) | 60 (29.13%) | 9 (47.37%) | 0.023 |
| **Type of ACS** |  |  |  |  | 0.163 |
| NSTE-ACS | 60 (43.8%) | 130 (37.9%) | 68 (33.01%) | 5 (26.32%) |  |
| STEMI | 77 (56.2%) | 213 (62.1%) | 138 (66.99%) | 14 (73.68%) |  |
| **Killip class >= II** | 56 (40.88%) | 183 (53.35%) | 124 (60.19%) | 17 (89.47%) | <0.001 |
| **WBC (x10^9^/L)** | 9.22 (7.61,11.65) | 9.54 (7.54,12.26) | 10.3 (7.31,13.93) | 11.45 (7.47,15.37) | 0.449 |
| **Hb (g/L)** | 129 (116,142) | 120 (105,133.5) | 105 (91,123) | 93 (82,105.5) | <0.001 |
| **Platelet (x10^9^/L)** | 210 (177,260) | 205 (167.5,249.5) | 191 (152.25,245.75) | 200 (142,231.5) | 0.009 |
| **Lymphocyte (x10^9^/L)** | 1.83 (1.53,2.2) | 1.31 (1.01,1.7) | 0.94 (0.7,1.2) | 0.69 (0.44,0.8) | <0.001 |
| **Creatinine (mg/dL)** | 1.41 (1.27,1.67) | 1.49 (1.27,2.05) | 1.67 (1.32,3.18) | 1.7 (1.48,2.64) | <0.001 |
| **eGFR (mL/min/1.73m^2^)** | 48.77 (38.03,54.86) | 44.2 (29.62,53.36) | 37.22 (16.55,50.5) | 36.99 (23.79,44.72) | <0.001 |
| **TC (mg/dL)** | 198.66 (173.64,224.84) | 175.94 (148.61,207.13) | 160.35 (128.98,186.63) | 112.81 (92.98,124.16) | <0.001 |
| **Albumin (g/L)** | 37.7 (36.3,39.5) | 34.8 (32.9,37.3) | 30.7 (28.92,33.18) | 25.6 (23.85,27.85) | <0.001 |
| **CRP (mg/L)** | 11.6 (5,28.5) | 20.7 (8.1,50.9) | 34.25 (14.35,73.65) | 66.6 (48.4,90) | <0.001 |
| **FBG (mmol/l)** | 6.3 (5.27,8.3) | 7 (5.5,9.3) | 7.2 (5.8,10.4) | 7.1 (5.25,8.1) | 0.012 |
| **LVEF < 40%** | 14 (10.22%) | 78 (22.74%) | 63 (30.58%) | 4 (21.05%) | <0.001 |
| **Multivessel disease** | 52 (37.96%) | 160 (46.65%) | 90 (43.69%) | 7 (36.84%) | 0.333 |
| **LAD stenosis >= 50%** | 108 (78.83%) | 286 (83.38%) | 168 (81.55%) | 15 (78.95%) | 0.682 |
| **LCX stenosis >= 50%** | 92 (67.15%) | 224 (65.31%) | 130 (63.11%) | 11 (57.89%) | 0.795 |
| **RCA stenosis >= 50%** | 89 (64.96%) | 257 (74.93%) | 155 (75.24%) | 14 (73.68%) | 0.129 |
| **DAPT** | 134 (97.81%) | 340 (99.13%) | 205 (99.51%) | 19 (100%) | 0.434 |
| **Beta blocker** | 83 (60.58%) | 191 (55.69%) | 88 (42.72%) | 5 (26.32%) | <0.001 |
| **ACEI / ARB** | 55 (40.15%) | 99 (28.86%) | 52 (25.24%) | 5 (26.32%) | 0.026 |
| **Statin** | 134 (97.81%) | 310 (90.38%) | 180 (87.38%) | 14 (73.68%) | 0.001 |
| **GRACE risk score** | 124 (107,138) | 134 (121,148) | 142.5 (128,158) | 148 (139,159) | <0.001 |

Abbreviations as in Table 1.
