# Supplementary material for: Prevalence and prognostic value of malnutrition in patients with acute coronary syndrome and chronic kidney disease
Source: Front Nutr. 2023 Jul 14;10:1187672. doi: 10.3389/fnut.2023.1187672 (PMC10376694; doi:10.3389/fnut.2023.1187672)
Supplement: Supplementary file 3 [file Table_3.DOCX]

Supplement table 3. Baseline characteristics by GNRI categories

| **Variables** | **GNRI** | | | | **P-value** |
| --- | --- | --- | --- | --- | --- |
|  | **≥ 100**  **Normal**  **(n=72)** | **97.5-99.9**  **Mild**  **(n=66)** | **83.5-97.4**  **Moderate**  **(n=470)** | **<83.5**  **Severe**  **(n=97)** |  |
| **Age (years)** | 67 (58,76) | 72 (65,81) | 74 (66,80) | 74 (65,81) | 0.003 |
| **Male** | 63 (87.5%) | 55 (83.33%) | 346 (73.62%) | 64 (65.98%) | 0.004 |
| **Height (cm)** | 164.5 (160,168) | 165.3 (162,168) | 165 (158,168) | 164 (156,165.3) | 0.027 |
| **Weight (kg)** | 66.5 (60,71.88) | 65.5 (64.15,72) | 63 (56,68) | 55 (48,62.5) | <0.001 |
| **BMI (kg/m^2^）** | 24.58 (23.55,26.51) | 24.44 (23.31,26.72) | 23.41 (21.46,25.21) | 20.45 (18.9,23.64) | <0.001 |
| **Hypertension** | 62 (86.11%) | 53 (80.3%) | 362 (77.02%) | 69 (71.13%) | 0.129 |
| **Diabetes** | 27 (37.5%) | 24 (36.36%) | 179 (38.09%) | 49 (50.52%) | 0.127 |
| **Hyperlipidemia** | 48 (66.67%) | 37 (56.06%) | 234 (49.79%) | 45 (46.39%) | 0.033 |
| **Dialysis** | 5 (6.94%) | 2 (3.04%) | 45 (9.57%) | 15 (15.46%) | 0.01 |
| **Prior myocardial infarction** | 3 (4.17%) | 1 (1.52%) | 9 (1.91%) | 1 (1.03%) | 0.512 |
| **Prior PCI** | 9 (12.5%) | 3 (4.55%) | 26 (5.53%) | 3 (3.09%) | 0.059 |
| **Prior CABG** | 1 (1.39%) | 1 (1.52%) | 5 (1.06%) | 0 (0%) | 0.532 |
| **Smoking** | 30 (41.67%) | 32 (48.48%) | 171 (36.38%) | 29 (29.9%) | 0.087 |
| **Type of ACS** |  |  |  |  | 0.006 |
| NSTE-ACS | 34 (47.22%) | 33 (50%) | 170 (52.17%) | 26 (26.8%) |  |
| STEMI | 38 (52.78%) | 33 (50%) | 300 (63.83%) | 71 (73.2%) |  |
| **Killip class >= II** | 28 (38.89%) | 30 (45.45%) | 255 (54.26%) | 67 (69.07%) | 0.001 |
| **WBC (x10^9^/L)** | 8.4 (6.58,11.38) | 8.73 (7.62,11.45) | 9.77 (7.56,12.5) | 11.1 (8,15.37) | 0.015 |
| **Hb (g/L)** | 132 (114.75,149.25) | 128.5 (118.25,139) | 116.5 (101,132) | 103 (90,122) | <0.001 |
| **Platelet (x10^9^/L)** | 206 (174,236) | 189 (162,234) | 203 (166,253) | 215 (155,265) | 0.415 |
| **Lymphocyte (x10^9^/L)** | 1.52 (1.17,2.12) | 1.4 (1.03,1.85) | 1.29 (0.9,1.67) | 1.06 (0.83,1.41) | <0.001 |
| **Creatinine (mg/dL)** | 1.48 (1.3,2) | 1.45 (1.22,1.63) | 1.5 (1.28,2.01) | 1.9 (1.31,3.4) | 0.001 |
| **eGFR (mL/min/1.73m^2^)** | 48.88 (30.36,55) | 48.83 (38.92,55.22) | 42.9 (28.63,52.62) | 29.73 (15.4,50.95) | <0.001 |
| **TC (mg/dL)** | 179.02 (163.24,219.06) | 178.83 (144.28,217.43) | 174.4 (146.49,205.4) | 160.93 (125.12,199.43) | 0.027 |
| **Albumin (g/L)** | 40.65 (39.68,42.23) | 38.4 (37.95,38.9) | 34 (32.02,35.8) | 27.6 (25.4,29.7) | <0.001 |
| **CRP (mg/L)** | 16.85 (5,44.3) | 9.5 (5.4,37.2) | 22.8 (8.85,55.65) | 41.9 (19.4,80) | <0.001 |
| **FBG (mmol/l)** | 6.3 (5.35,8.1) | 6.9 (5.73,8.38) | 6.8 (5.47,9.33) | 7.05 (5.5,11.1) | 0.266 |
| **LVEF < 40%** | 17 (23.61%) | 8 (12.12%) | 103 (21.91%) | 31 (31.96%) | 0.027 |
| **Multivessel disease** | 25 (34.72%) | 26 (39.39%) | 213 (45.32%) | 45 (46.39%) | 0.304 |
| **LAD stenosis >= 50%** | 57 (79.17%) | 52 (78.79%) | 385 (81.91%) | 83 (85.57%) | 0.644 |
| **LCX stenosis >= 50%** | 41 (56.94%) | 38 (57.58%) | 317 (67.45%) | 61 (62.89%) | 0.168 |
| **RCA stenosis >= 50%** | 51 (70.83%) | 48 (72.73%) | 341 (72.55%) | 75 (77.32%) | 0.767 |
| **DAPT** | 71 (98.61%) | 65 (98.48%) | 465 (98.94%) | 97 (100%) | 0.532 |
| **Beta blocker** | 41 (56.94%) | 46 (69.7%) | 246 (52.34%) | 34 (35.05%) | <0.001 |
| **ACEI / ARB** | 25 (34.72%) | 23 (34.85%) | 141 (30%) | 22 (22.68%) | 0.263 |
| **Statin** | 68 (94.44%) | 63 (95.45%) | 426 (90.64%) | 81 (83.51%) | 0.033 |
| **GRACE risk score** | 123 (99,139) | 132 (119,145) | 136 (122,153) | 141 (127,156) | <0.001 |

Abbreviations as in Table 1.
