# Supplementary material for: Prevalence and prognostic value of malnutrition in patients with acute coronary syndrome and chronic kidney disease
Source: Front Nutr. 2023 Jul 14;10:1187672. doi: 10.3389/fnut.2023.1187672 (PMC10376694; doi:10.3389/fnut.2023.1187672)
Supplement: Supplementary file 4 [file Table_4.DOCX]

Supplement table 4. Baseline characteristics by PNI categories

| **Variables** | **PNI** | | | **P-value** |
| --- | --- | --- | --- | --- |
|  | **>38**  **Normal**  **(n=495)** | **35-38**  **Moderate**  **(n=104)** | **<35**  **Severe**  **(n=106)** |  |
| **Age (years)** | 72 (64,79) | 75 (67,80) | 75 (69,81) | 0.013 |
| **Male** | 393 (79.39%) | 70 (67.31%) | 65 (61.32%) | <0.001 |
| **Height (cm)** | 165 (160,168) | 165 (158,167) | 162 (156,168) | 0.136 |
| **Weight (kg)** | 64 (56.6,70) | 60 (55,66) | 60 (53,65.75) | 0.009 |
| **BMI (kg/m^2^）** | 23.64 (21.67,25.55) | 23.3 (20.77,24.17) | 23.16 (20.47,24.96) | 0.016 |
| **Hypertension** | 397 (80.2%) | 72 (69.23%) | 77 (72.64%) | 0.023 |
| **Diabetes** | 178 (35.96%) | 46 (44.23%) | 55 (51.89%) | 0.006 |
| **Hyperlipidemia** | 275 (55.56%) | 44 (42.31%) | 45 (42.45%) | 0.006 |
| **Dialysis** | 33 (6.67%) | 19 (18.27%) | 15 (14.15%) | <0.001 |
| **Prior myocardial infarction** | 10 (2.02%) | 1 (0.96%) | 3 (2.83%) | 0.621 |
| **Prior PCI** | 34 (6.87%) | 6 (5.77%) | 1 (0.94%) | 0.061 |
| **Prior CABG** | 5 (1.01%) | 2 (1.92%) | 0 (0%) | 0.372 |
| **Smoking** | 199 (40.2%) | 36 (34.62%) | 27 (25.47%) | 0.015 |
| **Type of ACS** |  |  |  | 0.051 |
| NSTE-ACS | 199 (40.2%) | 32 (30.77%) | 32 (30.19%) |  |
| STEMI | 296 (59.8%) | 72 (69.23%) | 74 (69.81%) |  |
| **Killip class >= II** | 244 (49.29%) | 63 (60.58%) | 73 (68.87%) | <0.001 |
| **WBC (x10^9^/L)** | 9.54 (7.54,12.03) | 10.3 (8.04,15.39) | 10.13 (7.04,14.68) | 0.179 |
| **Hb (g/L)** | 123 (108,135) | 109.5 (98,124) | 99 (86.25,116.75) | <0.001 |
| **Platelet (x10^9^/L)** | 206 (169.5,254) | 190 (152.5,231) | 200.5 (154.25,252.25) | 0.03 |
| **Lymphocyte (x10^9^/L)** | 1.45 (1.11,1.9) | 1 (0.71,1.2) | 0.84 (0.6,1.06) | <0.001 |
| **Creatinine (mg/dL)** | 1.47 (1.27,1.84) | 1.61 (1.3,3.09) | 1.91 (1.39,3.25) | <0.001 |
| **eGFR (mL/min/1.73m^2^)** | 45.8 (32.34,54.03) | 39.16 (17.05,50.47) | 30.51 (15.23,47.48) | <0.001 |
| **TC (mg/dL)** | 176.72 (148.61,210.98) | 166.71 (144.66,203.18) | 169.98 (131.09,197.79) | 0.033 |
| **Albumin (g/L)** | 35.9 (33.9,38.2) | 31.45 (30.4,33) | 28 (25.92,29.67) | <0.001 |
| **CRP (mg/L)** | 17.8 (7.27,44.3) | 34.8 (13.15,73.18) | 45.7 (23.4,82.5) | <0.001 |
| **FBG (mmol/l)** | 6.7 (5.4,8.9) | 7.4 (5.6,10.3) | 7.3 (5.6,10.9) | 0.046 |
| **LVEF < 40%** | 98 (19.8%) | 32 (30.77%) | 29 (27.36%) | 0.023 |
| **Multivessel disease** | 213 (43.03%) | 46 (44.23%) | 50 (47.17%) | 0.735 |
| **LAD stenosis >= 50%** | 402 (81.21%) | 85 (81.73%) | 90 (84.91%) | 0.669 |
| **LCX stenosis >= 50%** | 315 (63.64%) | 73 (70.19%) | 69 (65.09%) | 0.444 |
| **RCA stenosis >= 50%** | 362 (73.13%) | 73 (70.19%) | 80 (75.47%) | 0.688 |
| **DAPT** | 488 (98.59%) | 104 (100%) | 106 (100%) | 0.223 |
| **Beta blocker** | 281 (56.77%) | 47 (45.19%) | 39 (36.79%) | <0.001 |
| **ACEI / ARB** | 161 (32.53%) | 26 (25%) | 24 (22.64%) | 0.065 |
| **Statin** | 462 (93.33%) | 88 (84.62%) | 88 (83.02%) | <0.001 |
| **GRACE risk score** | 132 (118,148) | 142 (127,154) | 143 (130,159) | <0.001 |

Abbreviations as in Table 1.
