# Supplementary material for: Prevalence and prognostic value of malnutrition in patients with acute coronary syndrome and chronic kidney disease
Source: Front Nutr. 2023 Jul 14;10:1187672. doi: 10.3389/fnut.2023.1187672 (PMC10376694; doi:10.3389/fnut.2023.1187672)
Supplement: Supplementary file 5 [file Table_5.DOCX]

Supplemental Table 5. Univariable Cox regression analyses of variables predicting all-cause mortality

| **Variables** | **Univariable analysis** | |
| --- | --- | --- |
|  | **HR[95%CI]** | **P-value** |
| **Age (years)** | 1.06[1.04,1.08] | <0.001 |
| **Sex (female vs male)** | 0.65[0.47,0.91] | 0.013 |
| **BMI (kg/m^2^）** | 0.92[0.88,0.97] | 0.002 |
| **Hypertension** | 0.94[0.65,1.35] | 0.728 |
| **Diabetes** | 1.07[0.77,1.48] | 0.701 |
| **Hyperlipidemia** | 0.61[0.44,0.85] | 0.003 |
| **Prior myocardial infarction** | 0.64[0.2,2.01] | 0.443 |
| **Prior PCI** | 0.54[0.24,1.23] | 0.142 |
| **Prior CABG** | 2.4[0.76,7.55] | 0.133 |
| **Smoking** | 0.746[0.52,1.07] | 0.110 |
| **Type of ACS** |  |  |
| NSTE-ACS | Reference | reference |
| STEMI | 2.34[1.58,3.48] | <0.001 |
| **Killip class >= II** | 2.13[1.52,2.99] | <0.001 |
| **Creatinine (mg/dL)** | 1.03[0.97,1.1] | 0.371 |
| **LVEF < 40%** | 2.06[1.48,2.87] | <0.001 |
| **Multivessel disease** | 0.95[0.69,1.32] | 0.780 |
| **Beta blocker** | 0.3[0.21,0.43] | <0.001 |
| **ACEI / ARB** | 0.45[0.29,0.68] | <0.001 |
| **Statin** | 0.08[0.06,0.11] | <0.001 |
| **GRACE risk score** | 1.03[1.02,1.04] | <0.001 |
| **Nutrition status** |  |  |
| CONUT |  |  |
| Normal nutrition | reference | reference |
| Mild malnutrition | 4.4[1.76,10.98] | 0.001 |
| Moderate malnutrition | 11.4[4.61,28.16] | <0.001 |
| Severe malnutrition | 31.38[11.16,88.23] | <0.001 |
| PNI |  |  |
| Normal nutrition | reference | reference |
| Moderate malnutrition | 2.89[1.91,4.39] | <0.001 |
| Severe malnutrition | 5.03[3.5,7.23] | <0.001 |
| GNRI |  |  |
| Normal nutrition | reference | reference |
| Mild malnutrition | 1.18[0.38,3.67] | 0.771 |
| Moderate malnutrition | 2.9[1.27,6.62] | 0.011 |
| Severe malnutrition | 6.56[2.78,15.47] | <0.001 |

Abbreviations as in Tables 1 and 2.
