# Supplementary material for: Prevalence and prognostic value of malnutrition in patients with acute coronary syndrome and chronic kidney disease
Source: Front Nutr. 2023 Jul 14;10:1187672. doi: 10.3389/fnut.2023.1187672 (PMC10376694; doi:10.3389/fnut.2023.1187672)
Supplement: Supplementary file 6 [file Table_6.DOCX]

Supplemental table 6. Multivariate Cox regression analyses for cardiovascular outcomes

|  | **Multivariable Analysis** | | | | | |
| --- | --- | --- | --- | --- | --- | --- |
|  | **Cardiovascular death** | | **Re-infarction** | | **Stroke** | |
|  | **HR [95% CI]** | **P-value** | **HR [95% CI]** | **P-value** | **HR [95% CI]** | **P-value** |
| **CONUT (continuous)** | 1.13 [1.02,1.26] | 0.021 | 1.11 [0.96,1.29] | 0.157 | 1.17 [0.95,1.43] | 0.134 |
| **CONUT (categorical; normal nutrition as reference)** | | | | |  |  |
| Mild risk | 1.93 [0.57,6.52] | 0.288 | 0.86 [0.37,1.98] | 0.721 | 2.09 [0.58,7.51] | 0.259 |
| Moderate risk | 3.07 [0.88,10.75] | 0.08 | 1.6 [0.64,3.98] | 0.316 | 1.45 [0.3,6.97] | 0.641 |
| Severe risk | 4.11 [0.9,18.76] | 0.068 | 2.02 [0.22,18.89] | 0.539 | 26.42 [3.42,204.07] | 0.002 |
| **GNRI (continuous)** | 0.98 [0.94,1.01] | 0.155 | 0.98 [0.93,1.03] | 0.377 | 0.95 [0.89,1.02] | 0.176 |
| **GNRI (categorical; normal nutrition as reference)** | | | | |  |  |
| Mild risk | 0.83 [0.13,5.22] | 0.838 | 1.17 [0.23,5.98] | 0.850 | 1.2 [0.15,9.4] | 0.861 |
| Moderate risk | 1.88 [0.55,6.38] | 0.313 | 2.01 [0.59,6.89] | 0.267 | 1.34 [0.28,6.4] | 0.716 |
| Severe risk | 1.53 [0.42,5.59] | 0.522 | 2.33 [0.56,9.64] | 0.244 | 2.95 [0.49,17.68] | 0.235 |
| **PNI (continuous)** | 0.94 [0.9,0.98] | 0.004 | 0.93 [0.88,0.99] | 0.019 | 0.93 [0.86,1.01] | 0.073 |
| **PNI (categorical; normal nutrition as reference)** | | | | |  |  |
| Moderate risk | 1.81 [1.03,3.18] | 0.04 | 2.63 [1.27,5.42] | 0.009 | 1.2 [0.36,4.01] | 0.763 |
| Severe risk | 1.64 [0.93,2.87] | 0.086 | 1.95 [0.85,4.45] | 0.114 | 1.56 [0.49,4.97] | 0.450 |

Abbreviations as in Tables 1 and 2.
